# CD248‐expressing cancer‐associated fibroblasts induce non‐small cell lung cancer metastasis via Hippo pathway‐mediated extracellular matrix stiffness

**DOI:** 10.1111/jcmm.70025

**Published:** 2024-08-20

**Authors:** Jiangwei Wu, Qiaoling Zhang, Zeyang Yang, Yujun Xu, Xinlei Liu, Xuanying Wang, Jiangying Peng, Jing Xiao, Yun Wang, Zhenling Shang, Nianxue Wang, Long Li, Rui Zhang, Wei Zhang, Jian Zhang, Zhu Zeng, Jieheng Wu

**Affiliations:** ^1^ Department of Immunology Guizhou Medical University Guiyang China; ^2^ Department of Biology Guizhou Medical University Guiyang China; ^3^ Guizhou Prenatal Diagnsis Center The Affiliated Hospital of Guizhou Medical University Guiyang China; ^4^ Department of Pharmaceutical Analysis Zunyi Medical University Zunyi China; ^5^ Immune Cells and Antibody Engineering Research Center of Guizhou Province, Key Laboratory of Biology and Medical Engineering Guizhou Medical University Guiyang China; ^6^ Key Laboratory of Infectious Immune and Antibody Engineering of Guizhou Province, Engineering Research Center of Cellular Immunotherapy of Guizhou Province, School of Biology and Engineering/School of Basic Medical Sciences Guizhou Medical University Guiyang China; ^7^ Department of Thoracic Surgery The Affiliated Hospital of Guizhou Medical University Guiyang China; ^8^ Department of Biochemistry and Molecular Biology, The State Key Laboratory of Cancer Biology The Fourth Military Medical University Xi'an China; ^9^ Department of Biochemistry and Molecular Biology Jilin Medical University Jilin China; ^10^ Tumor Immunotherapy Technology Engineering Research Center Guizhou Medical University Guiyang China

**Keywords:** cancer‐associated fibroblasts, CD248, extracellular matrix stiffness, hippo pathway, metastasis, non‐small cell lung cancer

## Abstract

Metastasis is a crucial stage in tumour progression, and cancer‐associated fibroblasts (CAFs) support metastasis through their participation in extracellular matrix (ECM) stiffness. CD248 is a possible biomarker for non‐small cell lung cancer (NSCLC)‐derived CAFs, but its role in mediating ECM stiffness to promote NSCLC metastasis is unknown. We investigated the significance of CD248^+^ CAFs in activating the Hippo axis and promoting connective tissue growth factor (CTGF) expression, which affects the stromal collagen I environment and improves ECM stiffness, thereby facilitating NSCLC metastasis. In this study, we found that higher levels of CD248 in CAFs induced the formation of collagen I, which in turn increased extracellular matrix stiffness, thereby enabling NSCLC cell infiltration and migration. Hippo axis activation by CD248^+^ CAFs induces CTGF expression, which facilitates the formation of the collagen I milieu in the stromal matrix. In a tumour lung metastasis model utilizing fibroblast‐specific CD248 gene knockout mice, CD248 gene knockout mice showed a significantly reduced ability to develop tumour lung metastasis compared to that of WT mice. Our findings demonstrate that CD248^+^ CAFs activate the Hippo pathway, thereby inducing CTGF expression, which in turn facilitates the collagen I milieu of the stromal matrix, which promotes NSCLC metastasis.

## 1 INTRODUCTION

Lung cancer (LC) is highly prevalent among all cancers. Its prevalence and mortality rank second and first, respectively, among all malignant tumours. Non‐small cell lung cancer (NSCLC) constitutes ~80%–85% of newly diagnosed LC cases.^1^ Metastasis is the primary contributor to NSCLC‐associated mortality. Approximately 75% of NSCLC patients exhibit evidence of regional or distant metastasis, and only 15% of metastatic NSCLC patients live for at least 5 years after diagnosis.^2^ Exploring the molecular mechanisms underlying NSCLC metastasis remains the focus of cancer research.

Tumour metastasis has always been unavoidable in the field of cancer treatment. Therefore, the study of tumour metastasis is one of the most active areas of cancer research. Metastasis occurs when tumour cells invade the normal tissue surrounding the primary tumour and disseminate malignantly throughout the body.^3^ This diffusion necessitates the movement of malignant cells through the connective tissue and extracellular matrix (ECM) protein network.^4^, ^5^

The ECM is a complicated axis of macromolecules that surrounds the body's cells and can be subdivided into the basement membrane and interstitial matrix based on their functions.^6^ Fibroblasts are the primary producers of tumour‐specific ECM proteins in tumour tissues.^7^, ^8^ In addition to secreting components of the ECM, fibroblasts provide matrix tension, which facilitates sheet‐ and fibre‐collagen arrangements and regulates collagen alignment.^6^ Collagen is a ubiquitous protein and the main structural matrix component. Collagen I is the primary component of tumour connective tissue hyperplasia and is closely associated with the metastasis of numerous malignancies.^9^

As an essential component of the tumour microenvironment (TME), cancer‐associated fibroblasts (CAFs) can interact with other stromal or tumour cells and modulate immune evasion, drug resistance, and tumour angiogenesis.^10^ Tumour metastasis and other processes are essential, and CAFs can promote tumour metastasis by changing the ECM structure via increased collagen deposition, interlinking collagen fibres, remodelling the ECM, and inhibiting immune cell infiltration.^11^, ^12^, ^13^, ^14^, ^15^ Because of fibroblast diversity, CAFs have enhanced phenotypic heterogeneity and distinctive functions per phenotype; consequently, the potential molecular mechanism of CAFs in NSCLC tumour metastasis remains poorly understood.

Endosialin/tumour endothelial marker 1 (CD248) is a type I transmembrane glycoprotein. The majority of tumour neovascular endothelial cells express CD248, while normal vascular endothelial cells do not.^16^, ^17^, ^18^ CD248 is also strongly expressed in diverse sarcomas, neuroblastomas, skin cancers, breast cancers, and additional tumours.^19^, ^20^ In addition, activated fibroblasts express CD248, which is involved in controlling fibroblast proliferation and migration.^16^ In renal cell carcinoma, CD248^+^ CAFs infiltration may contribute to renal cell carcinoma progression and an immunosuppressive TME through cell‐ ECM interactions and metabolic regulation.^21^ In our previous study, we demonstrated that CD248 expression in NSCLC‐based CAFs was associated with the adverse patient outcomes and clinicopathological features of NSCLC, including tumour differentiation and staging, lymph node metastasis, and tumour‐node‐metastasis (TNM) staging.^22^ We also demonstrated that CD248^+^CAFs mediate M2‐polarized macrophage‐ or secreted IL‐8‐induced NSCLC chemoresistance to promote NSCLC progression.^22^, ^23^

Yes‐associated protein (YAP) is now widely considered an effector of the Hippo pathway. YAP has become increasingly recognized as a mechanosensory response to external and internal mechanical stimuli, and mechanical stimuli associated with ECM stiffness, cell morphology, and cytoskeletal tension are dependent on the mechanically activated transcriptional program of YAP.^24^, ^25^, ^26^ CAFs have been shown to promote ECM remodelling via Hippo pathway activation, thereby promoting tumour metastasis.^27^ Connective tissue growth factor (CTGF), an upstream YAP target gene, is scarcely expressed in normal tissues and has elevated levels in fibrotic and malignant tissues.^28^ CTGF regulates numerous physiological activities, namely, cell adhesion, proliferation, migration, differentiation, ECM production, and vascular formation.^29^, ^30^, ^31^ In addition, CTGF stimulates collagen deposition, and many studies have shown that abnormal collagen deposition is strongly correlated with tumour progression.^32^, ^33^ However, whether CD248^+^CAFs can promote collagen I deposition, enhance ECM stiffness, and promote metastasis and invasion in NSCLC by activating the Hippo pathway has not been clearly elucidated.

Herein, we demonstrated that CD248^+^ CAFs promoted collagen I formation, which in turn increased ECM stiffness, facilitating NSCLC cell invasion and migration both in vitro and in vivo. Functional investigations revealed that CD248^+^CAFs activate the Hippo pathway, thereby inducing CTGF expression, which in turn facilitates the collagen I milieu in the stromal matrix. Our study emphasizes the importance and potential mechanism of CD248^+^CAFs in NSCLC tumour metastasis and provides a new strategy for improving the survival time of NSCLC patients.

## 2 MATERIALS AND METHODS

### 2.1 Human tumour specimens

Human NSCLC samples and matched normal tissues were obtained from The Affiliated Hospital of Guizhou Medical University. Guizhou Medical University granted ethical permission for this study (approval number 2022LL‐49), and all subjects provided signed documentation of their informed consent.

### 2.2 Mice

Suzhou Cyagen Co., Ltd., produced mice with floxed cd248 or fsp‐1‐Cre. *cd248* ^fl/fl^ *fsp‐1* ^+/+^ (WT) and *cd248* ^fl/fl^ *fsp‐1* ^cre/+^ (cKO) mice were generated by crossing floxed cd248 mice with fsp‐1‐Cre mice. All animals were bred and maintained in pathogen‐free environments, kept on a 12 hour light/dark cycle at 22 ± 1°C and 55% ± 5% relative humidity, and given free access to a standard laboratory diet. All rodents were housed in individual cages, had a C57BL/6 genetic background, and were aged between 6–12 weeks during experimentation. Cre‐negative littermate mice served as controls. All animal protocols received ethical approval from Guizhou Medical University.

### 2.3 Cell lines and coculture assay

The human NSCLC cell lines A549 and NCI‐H460, as well as murine melanoma B16‐F10 and LC LLC cells, were obtained from the American Type Culture Collection (ATCC, Manassas, VA, USA) and were tested for a lack of Mycoplasma contamination via short tandem repeat (STR) profiling. The cells were grown in Roswell Park Memorial Institute (RPMI) 1640 medium or Dulbecco's Modified Eagle Medium/Nutrient Mixture F‐12 (DMEM/F12) (Gibco Life Technologies, Waltham, MA, USA) supplemented with 10% FBS and 1% penicillin–streptomycin (Invitrogen Life Technologies, Waltham, MA, USA) at 37°C in a 5% CO_2_ humid chamber. Fibroblasts were isolated and cultivated as previously described.^22^, ^34^ We generated CAFs‐sh‐CD248 cells with sustained CD248 deficiency via lentiviral incorporation.^22^

### 2.4 Assessment of migratory and invasive abilities

To explore the migratory and invasive abilities of these cells, conditioned medium (CM) from CAFs‐sh‐CON/CAFs‐sh‐CD248/CAFs‐CD248‐OE was used initially. Before conducting Transwell experiments, A549 and NCI‐H460 cells were plated in six‐well plates, exposed to different CMs, and cultured for 48 h. For the migration and invasion assays, CAFs‐sh‐CON/CAFs‐sh‐CD248/CAFs‐CD248‐OE cells were placed in the bottom Transwell chamber, and A549 and NCI‐H460 cells were placed in the top chamber and then were incubated for 48 h. We conducted three separate experiments and analysed the results.

### 2.5 Western blot

We used the following primary antibodies for the Western blot analysis: anti‐human CD248 (CST, #47948), anti‐FAP (Servicebio, #GB11096), anti‐α‐SMA (Servicebio, #GB11044), anti‐S100A4 (Abcam, #ab197896), anti‐podoplanin (Servicebio, #GB115443), anti‐vimentin (Servicebio, #11192), anti‐YAP/TAZ (CST, #8418), anti‐phospho‐YAP (Ser127) (CST, #13008), anti‐phospho‐YAP (S397) (CST, #13619), anti‐MOB1 (CST, #13730), anti‐phospho‐MOB1 (CST, #8699), anti‐LATS1 (CST, #3477), anti‐collagen I (Servicebio, #11022), anti‐CTGF (Santa Cruz, #sc365970), anti‐TEAD1 (Santa Cruz, #sc376113), anti‐TEAD4 (Santa Cruz, #sc101184), anti‐YAP (Santa Cruz, #sc101199) and anti‐GAPDH (Servicebio). Proteins were separated using sodium dodecyl sulfate–polyacrylamide gel electrophoresis (SDS–PAGE) and then transferred to polyvinylidene difluoride membranes, which were subsequently blocked for 2 h in 5% bovine serum albumin (BSA) prepared in Tris‐buffered saline with Tween (TBST) at room temperature (RT). Then, the membranes were incubated with primary and secondary horseradish peroxidase (HRP)‐conjugated antibodies (Servicebio, #GB23303; #GB23301). We conducted three separate experiments and analysed the results. The protein bands were visualized with multi‐image light cabinet filter positions.

### 2.6 Real‐time quantitative polymerase chain reaction (RT–qPCR)

Total RNA was isolated using a MiniBEST Universal RNA Isolation Kit (Takara, #9767, San Jose, CA, USA) and then converted to cDNA with PrimeScript RT Master Mix (Takara, #RR063A). qPCR was conducted via a TB Green Premix Ex Taq II Kit (Takara, #RR820). Table S1 details the overall procedure.

### 2.7 Immunofluorescence (IF)

The following antibodies were used for IF: anti‐human CD248 (CST, #47948), anti‐α‐smooth muscle actin (SMA) (Abcam, #ab5694), anti‐collagen I (Servicebio, #11022), anti‐CTGF (Santa Cruz, #sc365970), and anti‐YAP (Santa Cruz, #sc101199). We employed tyramide signal amplification (TSA) technology to evaluate CD248, α‐SMA, collagen I, YAP, and CTGF expression and colocalization in tumour tissue slices via multiple immunofluorescence (IF) labeling. First, the slides were blocked for 2 h in 5% BSA at RT and then for 15 min in 3% H_2_O_2_. Step 1 involved primary antibody exposure for 1 h overnight (ON) at 4°C. Step 2 involved a 50 min incubation with an HRP‐conjugated secondary antibody at RT, following by washing in TBST. In Step 3, the slides were exposed to TSA fluorophores for 10 min at RT. In Step 4, the samples were boiled in EDTA antigen retrieval buffer to remove the antibody‐TSA complex. Steps 1–4 were repeated for each antibody. Laser scanning confocal microscopy was used to counterstain the nuclei with DAPI.

Immunofluorescent cells were fixed for 15 min in 4% paraformaldehyde (PFA) at RT and permeabilized for 10 min in 0.2% Triton X‐100 in PBS. After 2 h of blocking in 5% BSA at RT, the cells were incubated with anti‐CD248 (1:300), anti‐CTGF (1:500), anti‐collagen I (1:400), and anti‐YAP (1:600) antibodies ON at 4°C. The cells were incubated for 1 h with Alexa Fluor‐conjugated secondary antibodies (Servicebio) at RT. PI was used to counterstain the nuclei, and laser scanning confocal microscopy was used to capture the images.

### 2.8 Biomechanical characteristic measurements

Atomic force microscopy (AFM) (BioScope Catalyst, Bruker, Billerica, MA, USA) was employed to assess the Young's modulus of the CAFs and NFs. Each group was tested in contact mode with 100 cells. The pyramid probe on the cantilever tip touched one cell. The cantilever spring constant was 0.02 N/m. We conducted three separate experiments and assessed the results. The data are expressed as the means ± SDs.

### 2.9 RNA sequencing and GO and KEGG pathway assessment

TRIzol reagent was used for total RNA isolation from control shRNA‐ or CD248 shRNA‐stably infected CAFs. After the mRNA was isolated from the total RNA with poly(T) oligo‐attached magnetic beads, it was reverse transcribed into first‐strand cDNA by adding fragmentation buffer, DNA Polymerase I and RNase H, and random hexamer primers. The cDNA fragment ends were ligated to sequencing adaptors. Next, PTM Bio Ltd. (Hangzhou, China) was used to sequence and verify the quality of the RNA‐Seq library using an Agilent Bioanalyzer 2100 system (Agilent Technologies). GO and KEGG analyses were performed using the Gene Ontology (GO) database (https://amigo.geneontology.org/amigo) and KEGG pathway database (https://www.kegg.jp/kegg/pathway.html), respectively.

### 2.10 Mouse tumour experiments

Guizhou Medical University approved this animal study. To mimic lung metastasis, 5 × 10^5^ LLC‐luciferase or B16‐F10‐luciferase cells were intravenously administered to age‐ and sex‐matched WT and cKO mice in 200 μL of PBS. The mice bearing tumours were administered the substrate (D‐luciferin), anaesthetised with isoflurane, and evaluated with an in vivo imaging system for fluorescence imaging. The data were assessed using LivingImage software. A549 cells and CAFs infected with sh‐CD248 or sh‐CON lentiviruses were injected into BALB/c nude mice (4–6 weeks, male, 18–20 g body weight). Tumour volume was computed as follows: V (mm^3^) = a × b^2^/2, where a and b represent the long and short diameters, respectively. At the end of the experiment, the mice were euthanized, and their tumours were photographed. Paraffin sections of tumour tissues were prepared for H&E and IF staining.

### 2.11 Coimmunoprecipitation (Co‐IP) assay

Co‐IP was conducted with a coimmunoprecipitation kit (Absin, #abs9649) following the manufacturer's instructions. The cell lysates were incubated with the indicated antibodies for 60 min, followed by incubation with secondary‐antibody‐bound beads ON at 4°C, and then washing with buffer and ultrapure water. YAP, CD248, and rabbit IgG antibodies (2 μg per 1 mg of total protein) were used for the immunoprecipitation experiments.

### 2.12 Statistical analysis

The data were analysed using Excel 2016. Normality and equality tests of variance were performed on the quantitative data before analysis. The data from independent experiments are shown as the mean ± SD or the mean ± SEM. *P* values were calculated using two‐sided t tests. Differences were considered statistically significant at *p ≤ 0.05, **p < 0.01, ***p < 0.001 and ****p < 0.0001.

## 3 RESULTS

### 3.1 CD248 expression is evident on NSCLC‐based CAFs

**FIGURE 1:**
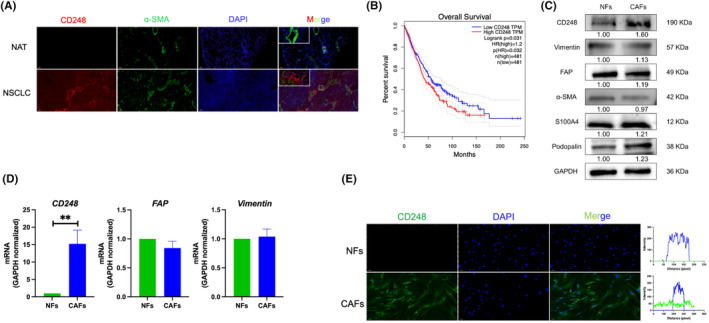
CAFs from NSCLC tissues strongly express CD248. (A) Representative dual immunofluorescence (IF) image demonstrating CD248 and α‐SMA colocalization in human NSCLC and normal adjacent tissues (NATs). Scale bar, 500 μm; upper panel, 20 μm. (B) Overall survival of patients with NSCLC from the GEPIA database. (C) Contents of CD248 and other epithelial biomarkers in extracted CAFs and NFs, as determined by Western blotting. (D) qPCR analysis demonstrating the contents of *CD248*, *FAP* and *vimentin* in CAFs and NFs. We conducted three separate experiments, and the results were analysed. The data are presented as the means ± SDs. ** *p* < 0.01. (E) IF staining demonstrating CD248 expression in CAFs and NFs. Scale bar, 50 μm.

To evaluate the expression profile of CD248 in NSCLC, immunofluorescence (IF) assays were performed on NSCLC and normal tissues. Positive expression of CD248 was observed in NSCLC tissues (Figure 1A); however, only weak expression was observed in nonneoplastic tissues (Figure 1A). CD248 was predominantly present in the CAFs of NSCLC tissues, as indicated by simultaneous α‐SMA colocalization (Figure 1A). NSCLC patients with higher CD248 levels had shorter survival times than patients with lower CD248 levels according to the Gene Expression Profiling Interactive Analysis (GEPIA) database (http://gepia.cancer‐pku.cn/) (Figure 1B). Next, we extracted fibroblasts from CAFs and adjoining NFs derived from NSCLC patients. Western blotting confirmed that the extracted CAFs and NFs expressed FAP, vimentin, α‐SMA, podoplanin and S100A4; however, the expression of CD248 was significantly greater in the CAFs than in the NFs (Figure 1C). RT–qPCR confirmed that the extracted CAFs and NFs expressed FAP and vimentin; however, the expression of CD248 was significantly greater in the CAFs than in the NFs (*p <* 0.01) (Figure 1D). The IF staining results demonstrated that fluorescence was quite visible among CAFs but not among NFs (Figure 1E). Based on these findings, CD248 is ubiquitous in CAFs derived from NSCLC tissues.

### 3.2 Collagen I generated by CD248‐expressing CAFs mediates ECM stiffness

**FIGURE 2:**
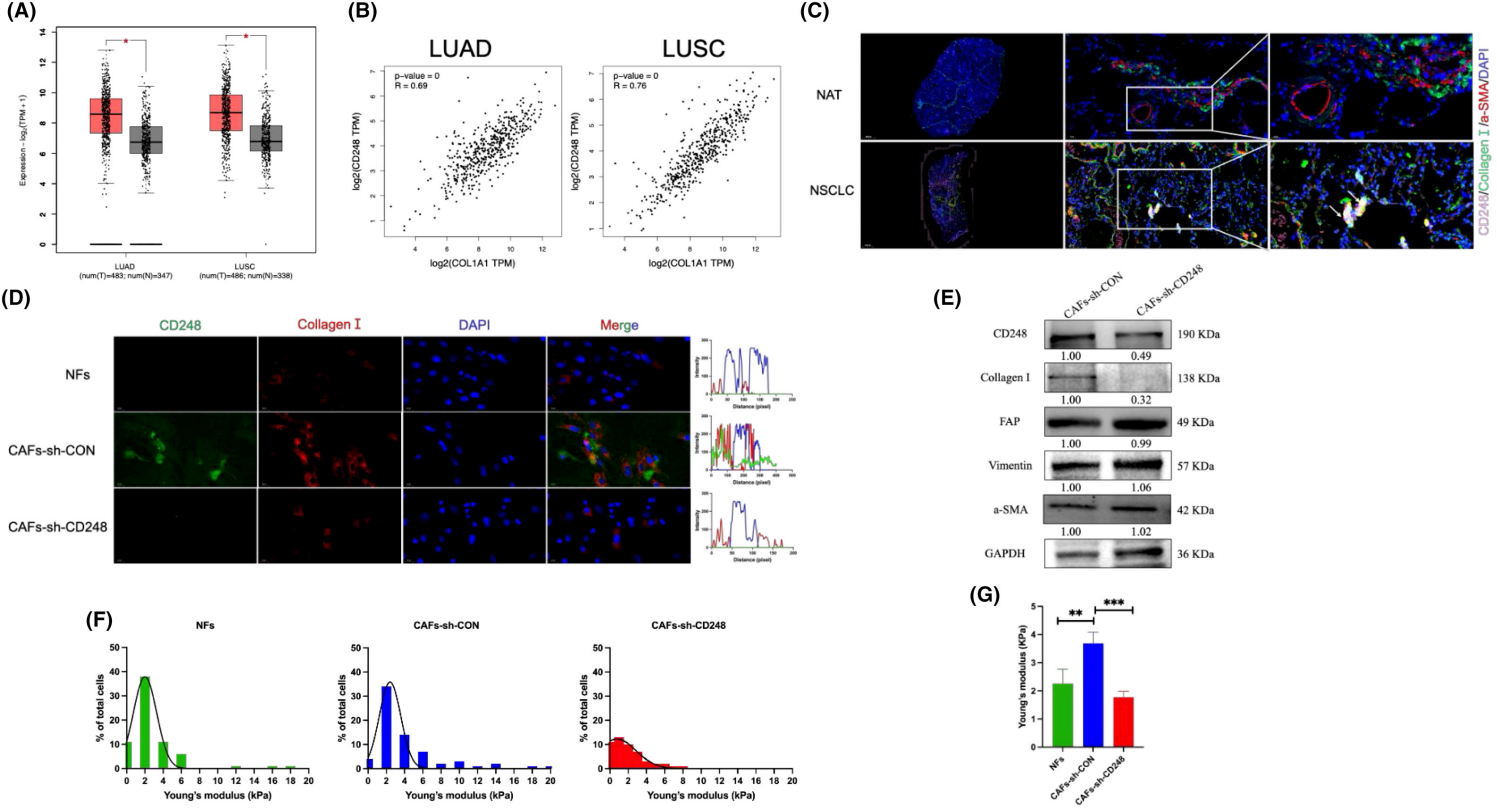
CD248‐expressing CAFs remodel the environment of stromal collagen I and subsequently promote ECM stiffness. (A) *COL1A1* expression profiles in LUAD tumour (*n* = 483) and normal (*n* = 347) tissues and LUSC tumour (*n* = 486) and normal (*n* = 338) tissues. (B) Relationship between *CD248* and *COL1A1* expression in LUAD and LUSC tumour tissues. (C) Images illustrating collagen I, CD248, and α‐SMA colocalization in human NSCLC tissues and NATs using dual IF staining. Scale bar, left: 2000 μm, middle: 50 μm; right: 20 μm. (D) IF staining showing collagen I and CD248 colocalization in NFs, CAFs‐sh‐CON, and CAFs‐sh‐CD248. Scale bar, 50 μm. (E) The expression of CD248, collagen I, and other epithelial markers in CAFs‐sh‐CON and CAFs‐sh‐CD248, as detected by Western blotting. (F) Young's modulus of NFs, CAFs‐sh‐CON, and CAFs‐sh‐CD248 to characterize mechanophenotypes and reflect ECM stiffness. (G) Statistical analysis of Young's modulus values of NFs, CAFs‐sh‐CON, and CAFs‐sh‐CD248. We conducted three separate experiments, and the results were analysed. The data are presented as the means ± SDs. *, *p* < 0.05; **, *p* < 0.01; ***, *p* < 0.001.

We used the Gene Expression Profiling Interactive Analysis (GEPIA) database (http://gepia.cancer‐pku.cn/) to detect *COL1A1* gene expression (encoding collagen I) and revealed that *COL1A1* expression was elevated in LUAD and LUSC tumour tissues relative to that in controls (*p <* 0.05) (Figure 2A). To determine whether the gene expression of *COL1A1* was correlated with that of *CD248* in NSCLC, we analysed the association between CD248 and *COL1A1* using the GEPIA database. The findings revealed that *COL1A1* expression correlated with *CD248* expression in LUAD and LUSC tumour tissues relative to that in NAT tissues (Figure 2B). We then conducted IF to detect the CD248 and collagen I content in NSCLC and NAT tissues. Positive expression of collagen I was observed in NSCLC tissues, as demonstrated by its simultaneous colocalization with CD248 and α‐SMA (Figure 2C). To determine whether CD248^+^CAFs produced collagen I, we used IF staining to detect collagen I expression in CD248‐deficient CAFs, control CAFs, and NFs. The IF staining results clearly revealed collagen I and CD248 fluorescence in the CAFs‐sh‐CON group but not in the CD248‐knockdown CAFs or NFs (Figure 2D). Western blot analysis confirmed that CAFs‐sh‐CD248 cells generated less collagen I than CAFs‐sh‐CON cells (Figure 2E). These results demonstrate that collagen I generation is associated with CD248^+^ CAFs.

To further characterize and elucidate the intrinsic characteristics of CD248‐expressing CAF‐generated collagen I‐mediated ECM stiffness, the mechanophenotypes of Young's modulus were used to assess potential alterations in CD248‐expressing CAFs. The findings demonstrated that the Young's modulus of CAFs was greater than that of NFs or CAFs‐sh‐CD248 (Figure 2F,G). This evidence demonstrates that CD248^+^ CAFs that generate collagen I promote ECM stiffness.

### 3.3 CD248 ^+^ CAFs facilitated NSCLC infiltration and migration in vitro and in vivo

**FIGURE 3:**
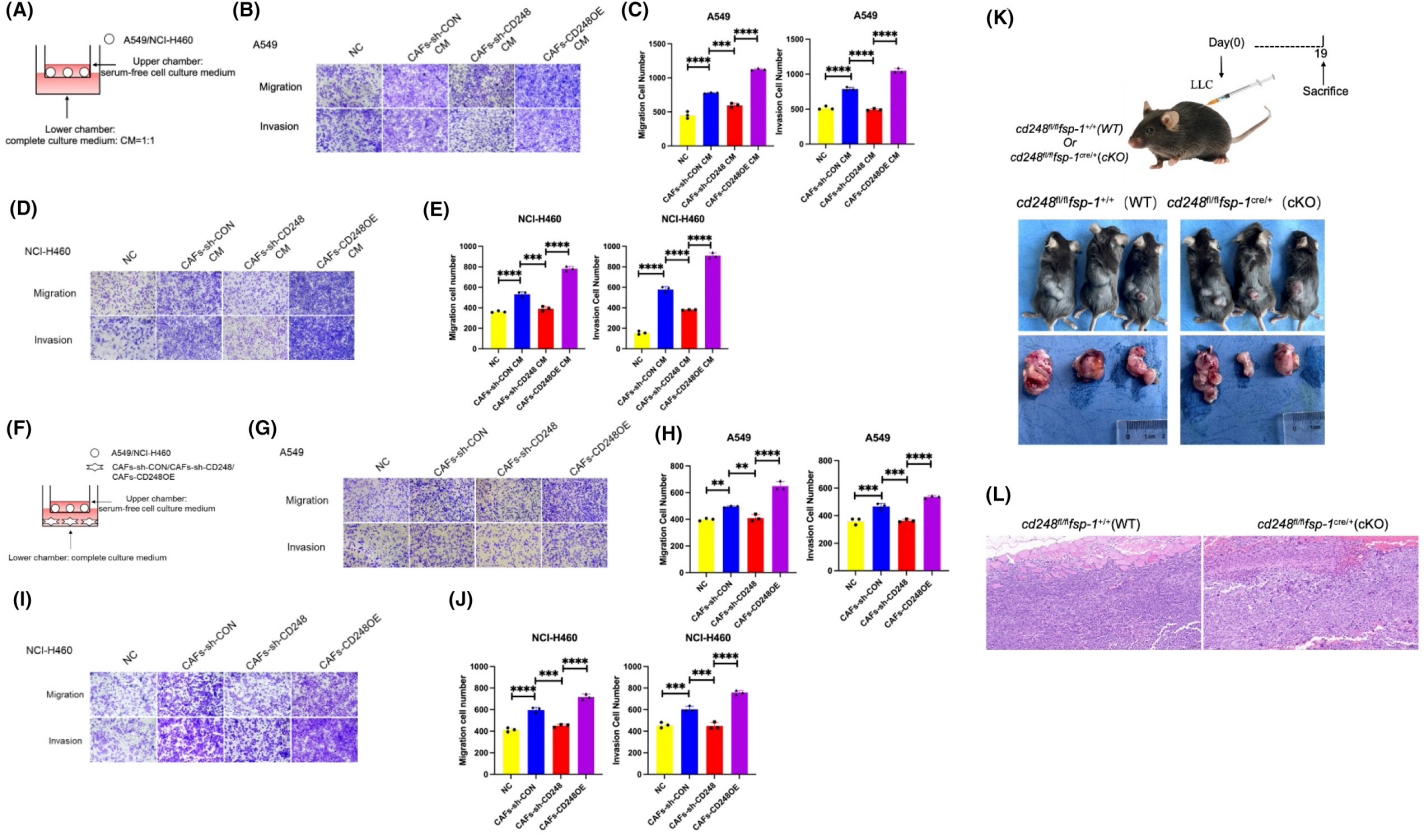
CD248‐expressing CAFs promote NSCLC migration and invasion. (A–E) NSCLC cell migration and invasion were characterized via Transwell assays. The NSCLC cell lines A549 and NCI‐H460 were cocultured with the CM of CAFs‐sh‐CON, CAFs‐sh‐CD248, or CAFs‐CD248OE. The CM alone (medium) served as the control in the coculture study (A). (B) After 24 h of coculture, A549 cell migration and invasion were evaluated. Scale bar, 200 μm. (C) The quantities of migratory and invasive cells were determined. We conducted three separate experiments. The data are presented as the means ± SDs. ***, *p* < 0.001; ****, *p* < 0.0001. (D) After 24 h of coculture, the migration and invasion abilities of NCI‐H460 cells were evaluated. Scale bar, 200 μm. (E) The quantities of migratory and invasive cells were calculated. We conducted three separate experiments. The data are presented as the means ± SDs. ***, *p* < 0.001; ****, *p* < 0.0001. (F–J) NSCLC cell migration and invasion were characterized via Transwell assays. A549 and NCI‐H460 cells were cocultured with CAFs‐sh‐CON, CAFs‐sh‐CD248, or CAFs‐CD248OE. CM alone (medium) served as a control in the coculture study (F). (G) After 24 h of coculture, the migration and invasion abilities of A549 cells were evaluated. Scale bar, 200 μm. (H) The migration and invasion abilities were calculated. We conducted three separate experiments. The data are presented as the means ± SDs. **, *p* < 0.01, ***, *p* < 0.001, ****, *p* < 0.0001. (I) After 24 h of coculture, the migration and invasion of NCI‐H460 cells were evaluated. Scale bar, 200 μm. (J) The quantities of migratory and invasive cells were calculated. We conducted three separate experiments. The data are presented as the mean ± SD. ***, *p* < 0.001; ****, *p* < 0.0001. (K, L) A total of 5 × 10^6^ LLC cells were injected subcutaneously into *cd248* ^fl/fl^ *fsp‐1* ^+/+^ (WT) and *cd248* ^fl/fl^ *fsp‐1* ^cre/+^ (cKO) mice to establish a xenograft models (*n* = 3 per group). (K) Diagram of the mouse model, whereby mice were euthanized and isolated tumour tissues were photographed. (L) Excised tumour tissues from each of the mouse groups were subjected to haematoxylin and eosin (H&E) staining to detect tumour metastasis. The white arrow indicates that the cancer cells had invaded the muscle layer.

To elucidate the significance of CD248^+^CAFs in NSCLC invasion and migration, the NSCLC cell lines A549 and NCI‐H460 were cocultured with CM derived from CAFs‐sh‐CON, CAFs‐sh‐CD248, or CAFs‐CD248OE, and then subjected to invasion and migration assays. Compared to the CAFs‐sh‐CD248 and control groups, the invasion and migration abilities of NSCLC cells cocultured with CAFs‐sh‐CON or CAFs‐CD248OE were significantly greater (*p* < 0.001) (Figure 3A–E). A549 and NCI‐H460 cells were cocultured with CAFs‐sh‐CON, CAFs‐sh‐CD248, or CAFs‐CD248OE before the invasion and migration assays were performed. Compared with the CAFs‐sh‐CD248 and control groups, the invasive and migratory abilities of A549 and NCI‐H460 cells cocultured with CAFs‐sh‐CON or CAFs‐CD248OE were substantially greater (*p < 0.001*) (Figure 3F–J).

To determine whether CD248^+^CAFs promote NSCLC invasion and migration in vivo, we subcutaneously injected LLC cells into *cd248* ^fl/fl^ *fsp‐1* ^+/+^ (WT) or *cd248* ^fl/fl^ *fsp‐1* ^cre/+^ (cKO) mice (Figure 3K). At the end of the experiment, the animals were euthanized, and the tissues were paraffin‐embedded to prepare them for haematoxylin–eosin (H&E) staining. The results demonstrated that the tumour cells invaded the muscle layer. In contrast, a relatively small number of tumour cells infiltrated the muscle layer of the CD248‐knockout mice (Figure 3L). These results indicated that CD248^+^CAFs enhanced the invasion and migration abilities of NSCLC cells.

### 3.4 CD248 may trigger ECM stiffness via activating the Hippo pathway based on the RNA sequencing and GO and KEGG database analyses

**FIGURE 4:**
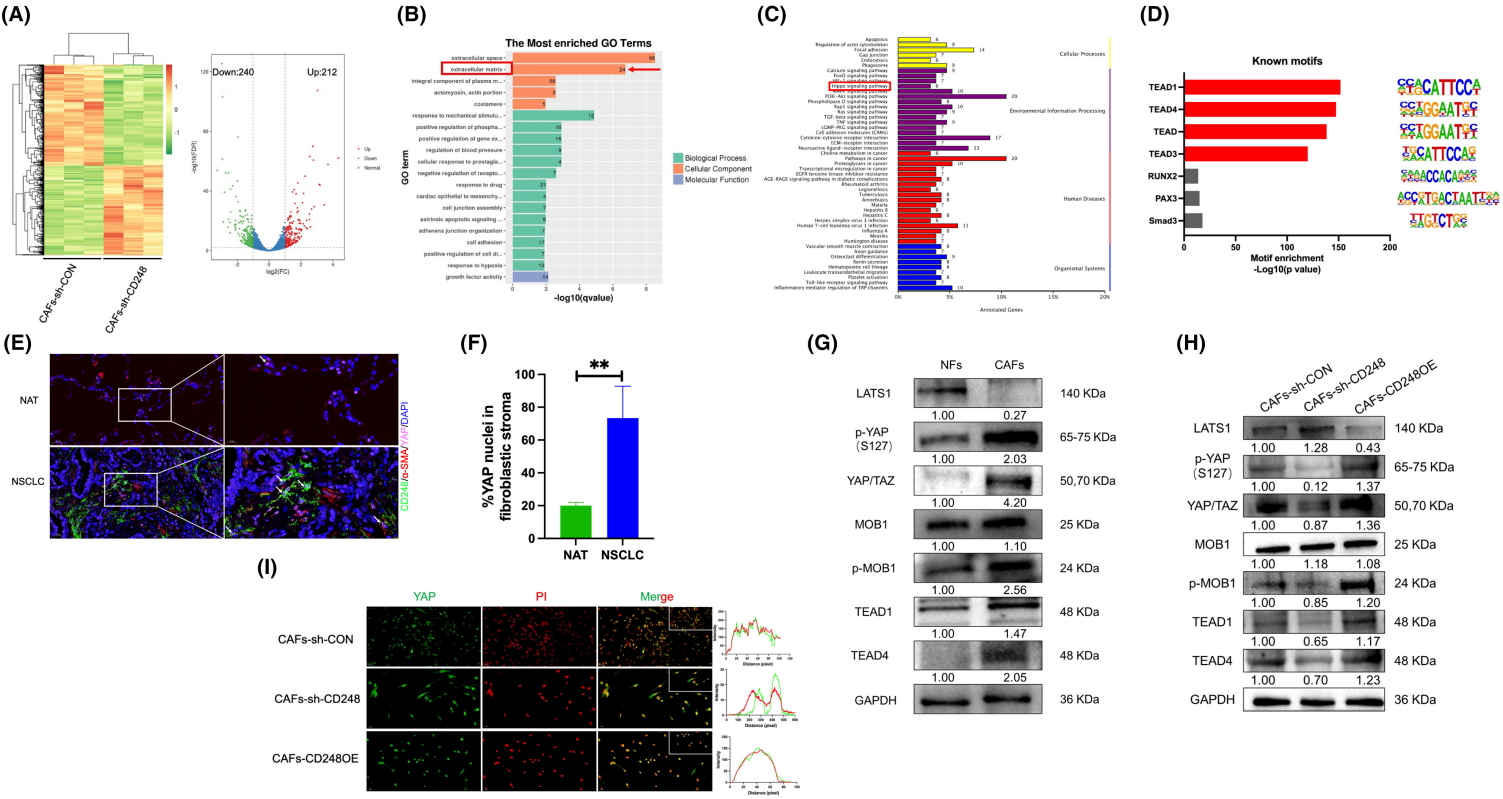
RNA sequencing of CD248^+^CAFs indicates the involvement of the Hippo pathway. (A) Heatmap showing distinct gene clustering in both control and CD248‐deficient CAFs. Volcano plots revealing the genes highly and scarcely expressed following CD248 knockdown. (B) GO analysis suggested that extracellular matrix networks contributed to the diminished networks following CD248 knockdown. (C) The top 20 networks whose expression decreased following CD248 knockdown, as determined by KEGG analysis. (D) Assay for transposase‐accessible chromatin using sequencing (ATAC‐Seq) techniques employing the CD248 CAFs open chromatin region‐enriched transcription factor. (E) IF staining revealing the colocalization of YAP, CD248, and α‐SMA in the NATs and NSCLC tissues. Scale bar: Left, 1000 μm; middle, 50 μm; right, 20 μm. (F) The number of YAP sites in the nuclei of the fibroblastic stroma in the NATs and NSCLC tissues. We conducted three separate experiments, and the results were analysed. The data are presented as the means ± SDs. **, *p* < 0.01. (G) Western blot analysis showing the levels of Hippo pathway proteins in NFs and CAFs. (H) Western blot analysis showing the levels of Hippo pathway proteins in CAFs‐sh‐CON, CAFs‐sh‐CD248, and CAFs‐CD248OE. (I) IF staining results showing that YAP was located in the nucleus of CAFs‐sh‐CON, CAFs‐sh‐CD248, and CAFs‐CD248OE. Scale bar, lower: 50 μm, upper: 20 μm.

We used CD248 shRNA for additional research because of its high CD248 knockdown efficiency and relatively stable cellular experimental performance. The heatmap displayed an altered gene cluster based on the RNA sequencing results (Figure 4A). Based on volcano plots, 240 genes were scarcely expressed (Figure 4A, labelled in green), and subsequent GO and KEGG network analyses suggested that these genes were intricately linked to multiple ECM networks, such as the Hippo pathway (Figure 4B,C). Using transposase‐accessible chromatin with high‐throughput sequencing (ATAC‐Seq) assays, we found that the open chromatin regions of CD248^+^CAFs were rich in docking regions for the transcription factors TEAD1 and TEAD4, which are Hippo pathway transcription factors regulating the ECM process (Figure 4D).

To determine whether CAFs induce ECM stiffness by activating the Hippo pathway, we examined YAP and CD248 expression and colocalization in NSCLC and NAT tissues using IF staining. The results demonstrated that in NSCLC tissues, the YAP fluorescence signal could be clearly detected in CD248^+^CAFs, and YAP translocated into the nucleus, whereas in NAT tissues, CD248 expression was not detected, and YAP was only slightly translocated into the nucleus (*p* < 0.01) (Figure 4E,F). We detected Hippo pathway protein expression in CAFs and NFs using Western blotting. Compared with those in CAFs, the levels of YAP, p‐YAP, MOB, p‐MOB, TEAD1, and TEAD4 were substantially reduced in NFs, whereas the levels of LATS1 and p‐LATS1 were markedly increased in NFs (Figure 4G).

YAP is negatively regulated by LATS1 and LATS2 phosphorylation, which in turn is negatively regulated by MST1 and MST2.^35^, ^36^ YAP phosphorylation is associated with cytoplasmic sequestration.^37^ Downregulated MST1/2 and LATS1/2 activity results in nuclear aggregation, TEAD and other protein binding, and transcriptional stimulation. To validate whether CD248^+^ CAFs induce ECM stiffness by activating the Hippo pathway, we examined Hippo pathway protein expression in CAFs‐sh‐CON, CAFs‐sh‐CD248, and CAFs‐sh‐CD248OE using Western blotting. YAP, p‐YAP, MOB, p‐MOB, TEAD1, and TEAD4 were expressed at significantly lower levels in CAFs‐sh‐CD248, whereas they were expressed at significantly greater levels in CAFs‐CD248OE (Figure 4H). Using IF staining, we confirmed that YAP was primarily found in the cytoplasm of CD248‐knockdown CAFs but translocated to the nucleus in CAFs and CAFs overexpressing CD248 (Figure 4I). These findings suggest that CD248^+^CAFs activate the Hippo pathway in NSCLC cells.

### 3.5 CD248 ^+^ CAFs activate the Hippo pathway, which induces CTGF expression to facilitate the collagen I milieu

**FIGURE 5:**
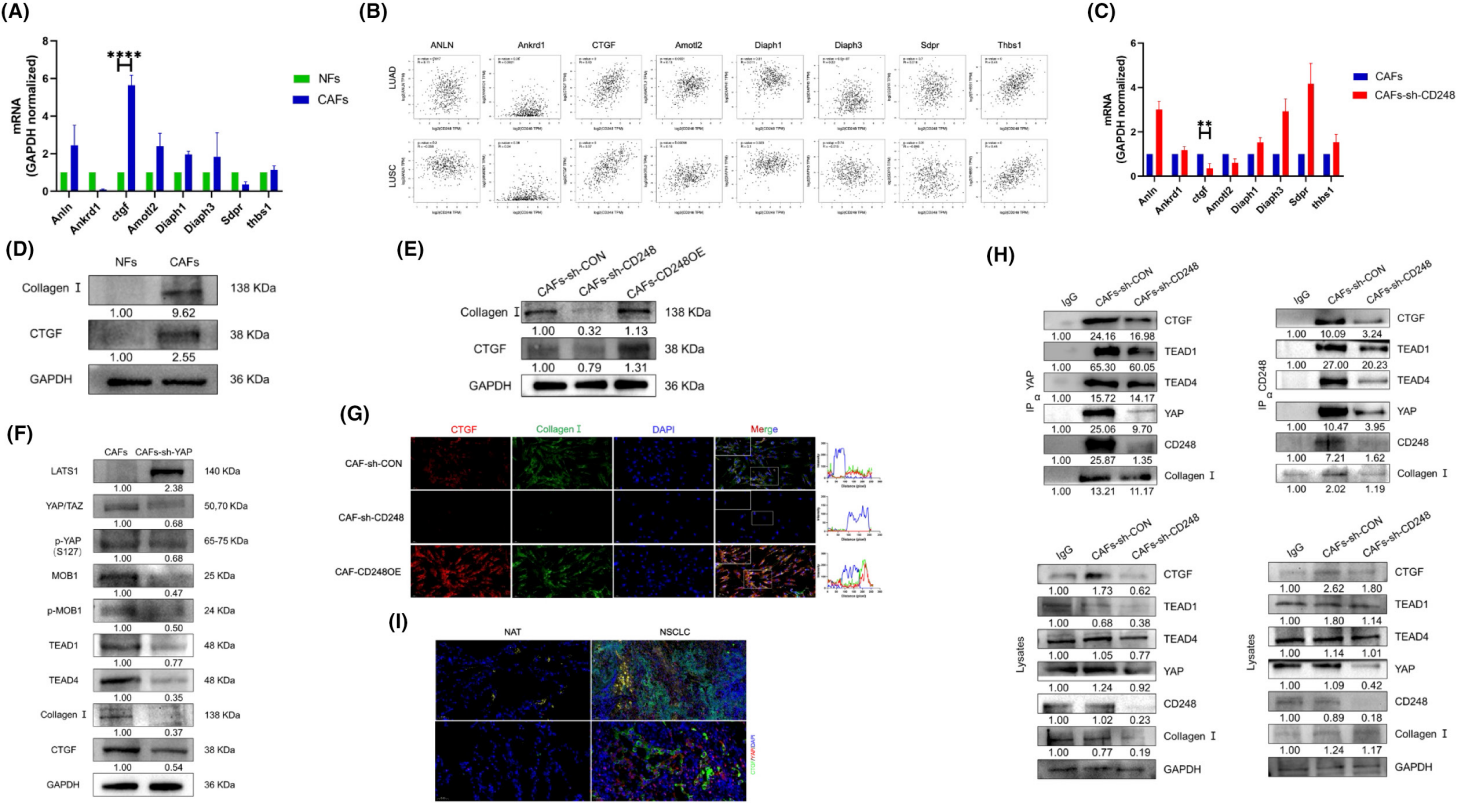
The CD248‐activated Hippo pathway induces CTGF expression‐mediated collagen I production by CAFs. (A) The gene expression of *ANLN*, *Ankrd1*, *CTGF*, *Amotl2*, *Diaph1*, *Diaph3*, *Sdpr* and *Thbs1* in NFs and CAFs was assessed by qPCR. We conducted three separate experiments, and the results were analysed. The data are presented as the means ± SDs. ****, *p* < 0.0001. (B) Relationships between *CD248* and *ANLN, Ankrd1, CTGF, Amotl2, Diaph1, Diaph3, Sdpr* and *Thbs1* expression in LUAD and LUSC tumour tissues. (C) *ANLN*, *Ankrd1*, *CTGF*, *Amotl2*, *Diaph1*, *Diaph3*, *Sdpr* and *Thbs1* levels in CAFs‐sh‐CON and CAFs‐sh‐CD248 were assessed by qPCR. We conducted three separate experiments, and the results were analysed. The data are presented as the means ± SDs. **, *p* < 0.01. (D) Western blotting data showing the CTGF and collagen I levels in NFs and CAFs. (E) Western blot data showing CTGF and collagen I levels in CAFs‐sh‐CON, CAFs‐sh‐CD248, and CAFs‐CD248OE cells. (F) Western blot data showing the levels of the Hippo pathway proteins, CTGF, and collagen I in the CAFs‐sh‐CON and CAFs‐sh‐YAP groups. (G) IF staining showing CTGF and collagen I colocalization in the CAFs‐sh‐CON, CAFs‐sh‐CD248, and CAFs‐CD248OE groups. Scale bar, 50 μm. (H) Coimmunoprecipitation was used to evaluate the interactions between CD248 and YAP, TEAD1, TEAD4, CTGF and collagen I in the indicated CAFs. (I) IF staining showing colocalization of CD248 (cyan), CTGF (green), collagen I (yellow) and YAP (red) in human NSCLC tissues and NATs. Scale bar, 50 μm.

*ANLN*, *Ankrd1*, *CTGF*, *AMOTL2*, *Diaph1*, *Diaph3*, *Sdpr* and *Thbs1* were recently shown to be transcriptionally modulated by YAP in CAFs. These genes are associated with ECM remodelling.^27^ We detected the expression of *ANLN*, *Ankrd1*, *CTGF*, *AMOTL2*, *Diaph1*, *Diaph3*, *Sdpr* and *Thbs1* in NFs and CAFs by qRT–PCR. The findings indicated that the *CTGF* content was substantially elevated in CAFs but not in NFs (*p* < 0.0001) (Figure 5A). Using the GEPIA database, we analysed the relationships between *CD248* and *ANLN*, *Ankrd1*, *CTGF*, *AMOTL2*, *Diaph1*, *Diaph3*, *Sdpr* and *Thbs1* in patients with LUAD and LUSC. The results revealed that the *CTGF* content was strongly correlated with the *CD248* level in LUAD and LUSC patients (Figure 5B). We detected the *ANLN*, *Ankrd1*, *CTGF*, *AMOTL2*, *Diaph1*, *Diaph3*, *Sdpr* and *Thbs1* levels in CAFs and CD248‐knockdown CAFs by qRT–PCR. *CTGF* expression in CAFs was substantially elevated relative to that in CD248‐knockdown CAFs (*p* < 0.01) (Figure 5C). Additionally, Western blot analysis revealed that the CTGF content was decreased in CD248‐knockdown CAFs and NFs (Figure 5D,E).

YAP and TEADs regulate CTGF expression, which enhances the collagen I milieu of ECM remodelling in bleomycin‐induced lung fibrosis.^38^ To verify whether the Hippo axis regulates CTGF expression, we constructed YAP‐knockdown CAFs and used Western blotting to evaluate the levels of the Hippo pathway proteins CTGF and collagen I. We revealed that the levels of YAP, p‐YAP, MOB, p‐MOB, TEAD1, TEAD4, CTGF and collagen I were significantly lower in CAFs‐sh‐YAP (Figure 5F).

IF staining was used to determine the relationship between CTGT and collagen I in CAFs‐sh‐CON, CAFs‐sh‐CD248, and CAFs‐CD248OE cells. CTGF was colocalized with collagen I in CAFs‐sh‐CON and CAFs‐CD248OE, and only a small amount of fluorescence was detected in CD248‐knockdown CAFs (Figure 5G). To verify whether CD248 can interact with YAP, CTGF, TEADs, and collagen I, we used co‐IP assays to determine the interaction between CD248, YAP, TEADs, CTGF, and collagen I. The results showed that YAP modulation occurred via association with TEAD1, TEAD4, CTGF, and collagen I in CD248^+^ CAFs. (Figure 5H). To detect the expression of CD248, collagen I, CTGF and YAP in NSCLC, we performed IF staining on NSCLC and nonneoplastic tissues. CD248, collagen I, CTGF and YAP were highly expressed, and YAP, CTGF and collagen I colocalized with CD248; a relatively faint fluorescent signal was detected in nonneoplastic tissues (Figure 5I). These results showed that CD248^+^CAFs activate the Hippo pathway, which induces CTGF expression to facilitate the collagen I milieu.

### 3.6 Fibroblast‐specific *CD248* depletion promoted LC metastasis in vivo

**FIGURE 6:**
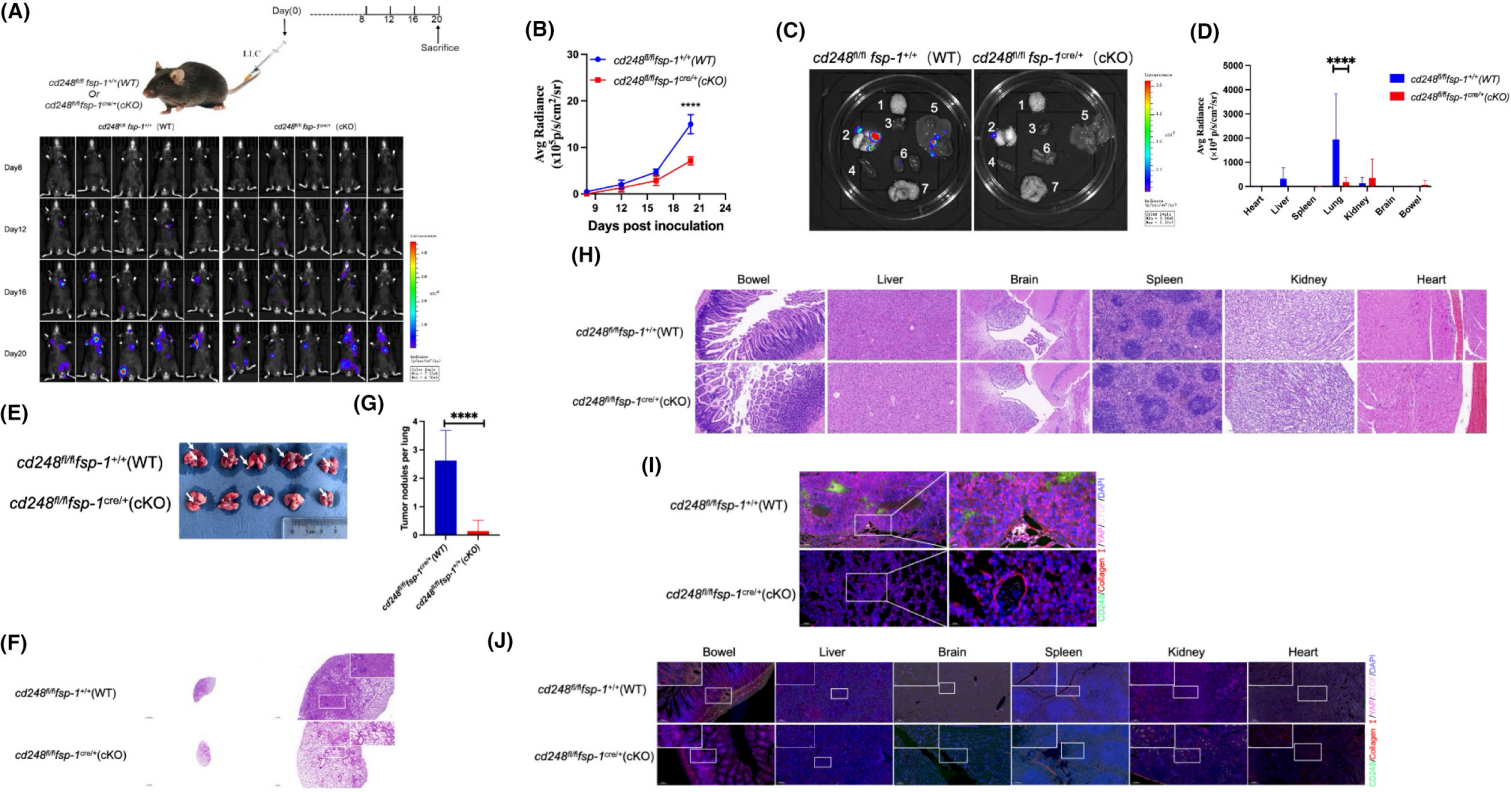
Fibroblast‐specific CD248 depletion inhibits lung cancer (LC) metastasis in vivo. A total of 5 × 10^5^ LLC cells were injected into *cd248* ^fl/fl^ *fsp‐1* ^+/+^ (WT) and *cd248* ^fl/fl^ *fsp‐1* ^cre/+^ (cKO) mice to establish an LC metastasis model (*n* = 5 per group). (A) An illustration of the murine model. Tumour development was monitored using BLI. (B) Fluorescence intensity assessment per cohort. The data are presented as the means ± SEMs. ****, *p* < 0.0001. (C) At the termination of the experiment, the mice were euthanized, and various tissues were extracted for BLI assessment: 1, brain; 2, lung; 3, heart; 4, spleen; 5, liver; 6, kidney and 7, bowel. (D) Fluorescence quantification of the various organs in C. The data are presented as the means ± SEMs. ****, *p* < 0.0001. (E) Lung tissues from each group of mice were isolated and photographed. The white arrow indicates the LC metastasis model. (F) H&E staining was used to detect lung tumour metastatic nodules. Scale bar, left: 2000 μm, right: 500 μm; upper: 100 μm. (G) Quantification of metastatic tumour nodules per lung. The data are presented as the means ± SEMs. ****, *p* < 0.0001. (H) Other organs of each group of mice were extracted for H&E staining to detect tumour metastasis nodules. Scale bar, 100 μm. (I) Dual‐IF staining results showing collagen I (red), CTGF (pink), YAP (rose red), and CD248 (green) colocalization in tumour metastasis nodules in the lungs of WT and cKO mice. Scale bar, left: 100 μm; right: 20 μm. (J) Dual‐IF staining results showing the colocalization of collagen I (red), CTGF (pink), YAP (red), and CD248 (green) in the other organs of WT and cKO mice. Scale bar, 100 μm; upper panel, 20 μm.

To determine whether CD248 affects tumour metastasis in vivo, we injected LLC cells into the tail veins of *cd248* ^fl/fl^ *fsp‐1* ^+/+^ (WT) and *cd248* ^fl/fl^ *fsp‐1* ^cre/+^ (cKO) mice (Figure 6A). We meticulously monitored tumour growth via bioluminescence imaging (BLI) and recorded the fluorescence intensity of the tumour. Our findings revealed that tumour metastasis was substantially greater in WT mice than in CD248‐deficient mice (Figure 6A,B). At the termination of the experiment, the mice were euthanized, and various tissues were extracted and analysed for their fluorescence intensities. In WT mice, we detected a strong fluorescence signal in the lung tissue, a relatively faint signal in the liver and intestine tissues, and a negligible signal in all other tissues. In contrast, in CD248‐deficient mice, only a relatively faint fluorescent signal was detected in the lungs, whereas no signal was detected in the other tissues (*p* < 0.0001) (Figure 6C,D). Lung and other tissues were harvested from euthanized mice, and paraffin sections were prepared for H&E staining. Compared to cKO mice, WT mice had significantly larger and more extensive lung metastatic nodules, as revealed by histological examination (*p* < 0.0001) (Figure 6E–G). Metastatic nodules were not detected in the other tissues of the WT and cKO mice (Figure 6H). Paraffin‐embedded tissues from euthanized mice were subjected to IF staining for CD248, collagen I, CTGF, and YAP. In the lung metastasis nodules of WT mice, CD248, collagen I, CTGF, and YAP were highly expressed, and YAP, CTGF, and collagen I colocalized with CD248; a relatively faint fluorescent signal was detected in the lung metastasis nodules of cKO mice (Figure 6I). CD248, collagen I, CTGF, and YAP were expressed at relatively low levels in the other tissues of the WT and cKO mice (Figure 6J). These results suggest that CD248‐expressing fibroblasts promote LC metastasis by activating the Hippo pathway, which induces CTGF expression to facilitate the collagen I milieu.

### 3.7 Fibroblast‐specific *CD248* depletion promoted B16 tumour lung metastasis in vivo

**FIGURE 7:**
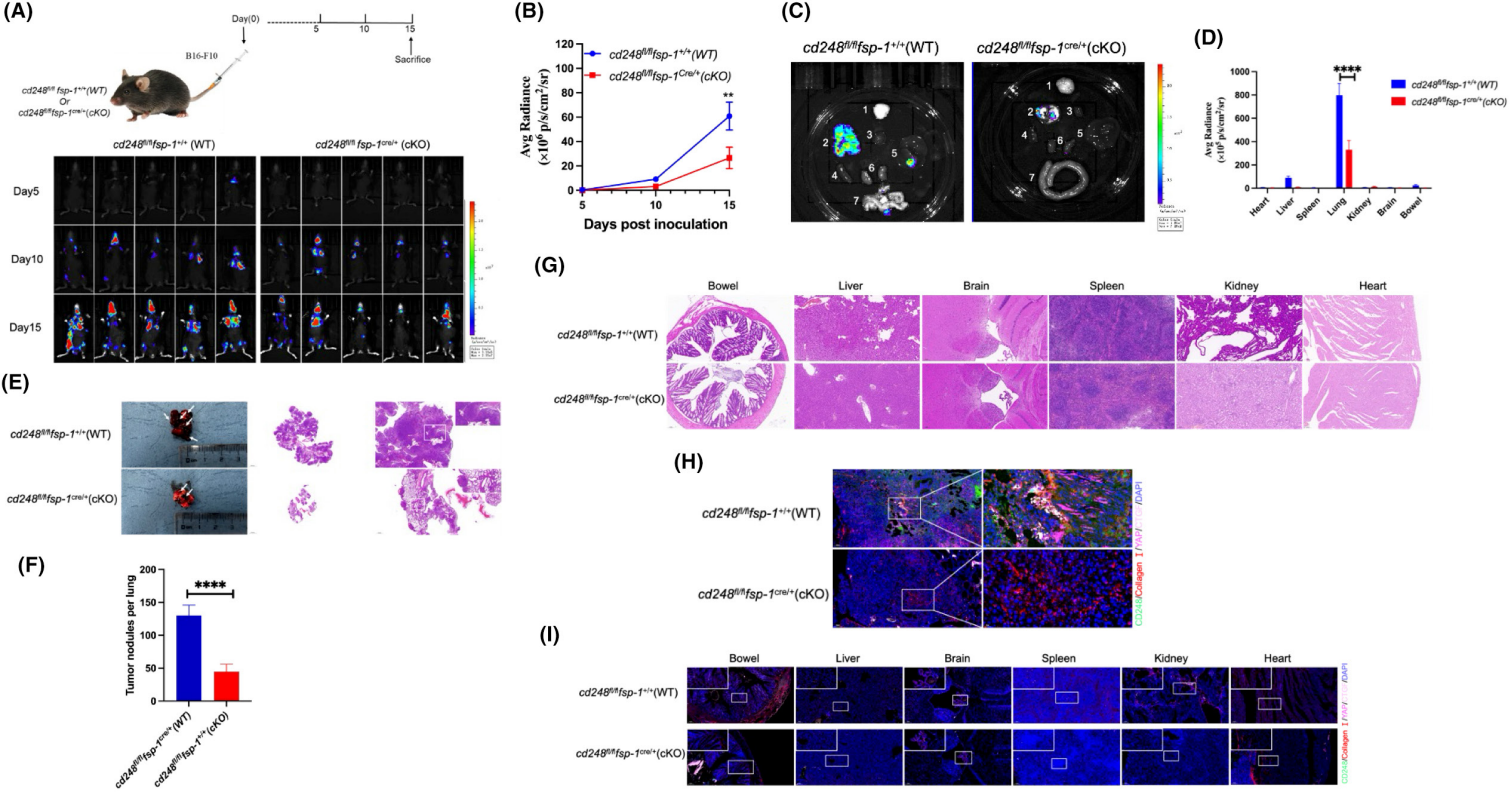
Fibroblast‐specific CD248 depletion inhibits B16‐F10 tumour lung metastasis. A total of 5 × 10^5^ B16‐F10 cells were injected into *cd248* ^fl/fl^ *fsp‐1* ^+/+^ (WT) and *cd248* ^fl/fl^ *fsp‐1* ^cre/+^ (cKO) mice to establish a melanoma lung metastasis model (*n* = 5 per group). (A) An illustration of the murine model. Tumour development was monitored using BLI. (B) Fluorescence intensity assessment per cohort. The data are presented as the means ± SEMs. **, *p* < 0.01. (C) At the termination of the experiment, the mice were euthanized, and distinct tissues were extracted for BLI assessment: 1, brain; 2, lung; 3, heart; 4, spleen; 5, liver; 6, kidney; and 7, bowel. (D) Fluorescence quantification of the various organs in C. The data are presented as the means ± SEMs. ****, *p* < 0.0001. (E) Lung tissues from each murine group were extracted for H&E staining to examine tumour metastasis nodules. The white arrow indicates the lung cancer (LC) metastasis model. Scale bar, left: 2000 μm, right: 500 μm; upper: 100 μm. (F) Quantification of the tumour metastasis nodules per lung. The data are presented as the means ± SEMs. ****, *p* < 0.0001. (G) Other organs from each murine group were extracted for H&E staining to detect tumour metastasis nodules. Scale bar, 100 μm. (H) Dual‐IF staining results showing collagen I (red), CTGF (pink), YAP (rose red), and CD248 (green) colocalization in the lung tumour metastasis nodules from WT and cKO mice. Scale bar, left: 100 μm; right: 20 μm. (I) Dual‐IF staining results showing the colocalization of collagen I (red), CTGF (pink), YAP (red), and CD248 (green) in the other organs of WT and cKO mice. Scale bar, 100 μm; upper panel, 20 μm.

**FIGURE 8:**
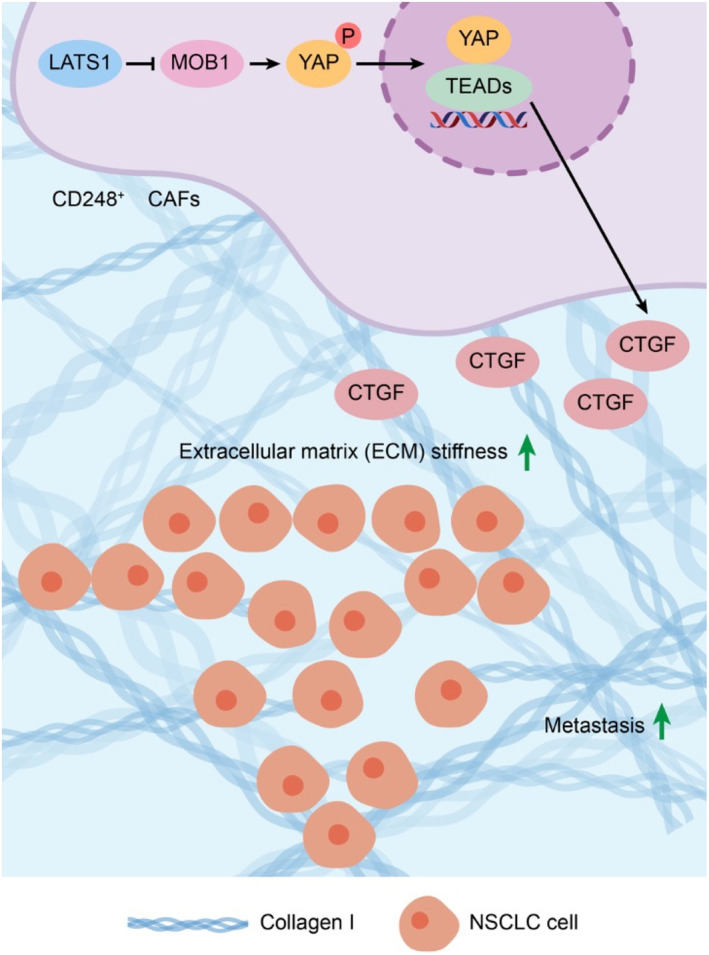
CD248^+^CAFs promote NSCLC metastasis via Hippo pathway‐mediated ECM stiffness. CD248^+^CAFs activate the Hippo pathway, resulting in an increase in CTGF expression, which in turn facilitates collagen I milieu remodelling of the stromal matrix and stiffening of the ECM, which promotes the metastasis of NSCLC.

B16‐F10 cells were injected intravenously into *cd248* ^fl/fl^ *fsp‐1* ^+/+^ (WT) or *cd248* ^fl/fl^ *fsp‐1* ^cre/+^ (cKO) mice to assess whether CD248 influences lung metastasis (Figure 7A). According to our results, tumour metastasis was substantially more frequent in WT mice than in CD248‐deficient mice (Figure 7A,B). Mice were euthanized at the termination of the experiment, various tissues were extracted, and fluorescence intensities were measured. The results indicated that the lung tissue of WT mice exhibited a significant fluorescence signal, whereas the liver and colon tissues exhibited relatively faint signals, and all other tissues exhibited no signal. In contrast, there was only a faint fluorescent signal in the lungs and no signal in the other organs in the CD248‐deficient mice (*p < 0.0001*) (Figure 7C,D). Lung and other tissues were harvested from sacrificed mice, and paraffin sections were prepared for H&E staining. Based on our histological evaluation, the WT mice exhibited larger and more extensive lung metastatic nodules than did the cKO mice (Figure 7E,F). Histological analysis demonstrated evidence of metastatic nodules in the liver tissue but not in the other tissues of the WT mice. In contrast, the cKO animals did not exhibit metastatic nodules in the other tissues (Figure 7G). Mouse tissues were processed into paraffin slices for immunofluorescence staining using antibodies against CD248, collagen I, CTGF, and YAP. CD248, collagen I, CTGF, and YAP were highly expressed, and YAP, CTGF, and collagen I colocalized with CD248 in the lung metastatic nodules of WT mice, whereas cKO mice exhibited a relatively faint fluorescent signal (Figure 7H). CD248, collagen I, CTGF, and YAP were not detected in the other tissues of either the WT or cKO mice (Figure 7I). These results demonstrated that CD248^+^fibroblasts promote B16‐F10 tumour lung metastasis by activating the Hippo pathway, which induces CTGF expression to facilitate the collagen I milieu related to ECM stiffness. In conclusion, our findings demonstrate that CD248^+^CAFs activate the Hippo pathway, thereby inducing CTGF expression, which in turn facilitates the collagen I milieu related to ECM stiffness, which promotes NSCLC metastasis (Figure 8).

## 4 DISCUSSION

LC is a dominant contributor to cancer‐related morbidity and mortality.^39^ LC can be classified histologically into two categories: small‐cell LC and NSCLC. NSCLC accounts for approximately 85% of all LC cases.^40^ Metastasis drives mortality among LC patients, and 40%–60% of LC patients exhibit metastases at diagnosis.^2^, ^41^, ^42^ Cancer cell metastasis is precisely regulated in vivo by environmental factors, including chemical and mechanical factors. Enhanced ECM stiffness promotes cancer cell metastasis.^43^, ^44^ CAFs are major TME components and play a critical role in promoting tumour progression by regulating ECM stiffness.^12^ The ECM is a complicated axis of macromolecules that surround cells within the body and can be subdivided into the basement membrane and interstitial matrix according to their functions.^6^ Alterations in the physical properties of the ECM in solid tumours can promote tumour cell migratory and invasive behaviours in interstitial tissue.^45^ CAFs can alter the structure of the ECM by promoting the deposition of collagen protein, mediating the cross‐linking of collagen fibres, causing ECM stiffness, inhibiting the infiltration of immune cells, and promoting tumour metastasis.^14^, ^15^ However, owing to the diversity of fibroblasts, CAFs exhibit increased phenotypic heterogeneity, and they possess distinct phenotypes with distinct functions.^10^ In our previous studies, we discovered that CD248 is a potential marker of CAFs derived from NSCLC.^22^ In this study, we discovered that CD248^+^CAFs induced the formation of collagen I, which increased ECM stiffness, thereby increasing the invasion and migration abilities of NSCLC cells. In a model of tumour lung metastasis, we also discovered that CD248 knockout mice had a significantly reduced ability to develop lung metastases compared to wild‐type mice.

It has been discovered that LOX‐induced collagen cross‐linking increases the rigidity of the ECM in mouse breast cancer models, thereby inducing an integrin‐reliant invasive phenotype.^46^ CAFs are the primary producers of tumour‐specific ECM components in tumour tissues.^7^, ^8^ In addition to secreting ECM components, CAFs are capable of exerting matrix tension, forming collagen tissue into sheets and filaments and influencing collagen arrangement.^7^ In a murine lung adenocarcinoma model, CAF‐expressed PLOD2‐mediated collagen cross‐linking increased tumour invasiveness.^47^ Mechanical signals activate YAP, a Hippo axis transcriptional coactivator that regulates the expression of cytoskeletal regulatory factors in CAFs, thereby increasing intracellular tension and promoting matrix sclerosis.^24^, ^48^ It was established that the epithelial‐to‐mesenchymal transition (EMT) factor SNAIL1 plays an essential role in ECM production in CAFs and influences its ability to increase matrix hardness.^49^, ^50^, ^51^ We first demonstrated that CD248^+^CAFs activate the Hippo pathway, resulting in an increase in CTGF expression. This facilitates remodelling of the collagen I environment of stromal matrix stiffness. These mechanisms promote NSCLC metastasis by facilitating ECM stiffness.

Our study demonstrated that CD248^+^CAFs activate the Hippo pathway, thereby inducing CTGF expression, which in turn facilitates the collagen I milieu of the stromal matrix, promoting the metastasis of NSCLC. However, this article contains unclear explanations. Insufficient research has been conducted on the regulation of YAP by CD248^+^CAFs, and the molecular mechanisms and interactions between CD248 and YAP have not yet been identified. Thus, our follow‐up research will focus on determining the precise molecular mechanism of this phenomenon, and we will investigate the precise molecular mechanism by which CD248 regulates YAP activity.

Herein, we present the significance of CD248‐expressing CAFs in inducing ECM stiffness and promoting NSCLC metastasis by activating the Hippo pathway. It also identifies CD248 as a novel biomarker for NSCLC‐based CAFs and a novel tool for targeting CD248‐harbouring CAFs to reverse NSCLC metastasis.

## AUTHOR CONTRIBUTIONS

**Jiangwei Wu:** Data curation (equal); formal analysis (equal); investigation (equal); supervision (equal); writing – original draft (lead). **Qiaoling Zhang:** Data curation (equal); formal analysis (equal); methodology (equal); visualization (equal). **Zeyang Yang:** Formal analysis (equal); investigation (equal); methodology (equal). **Yujun Xu:** Investigation (equal); project administration (equal). **Xinlei Liu:** Data curation (equal); project administration (equal); resources (equal). **Xuanying Wang:** Data curation (supporting); investigation (supporting); methodology (supporting). **Jiangying Peng:** Methodology (supporting); visualization (supporting). **Jing Xiao:** Investigation (supporting); project administration (supporting). **Yun Wang:** Data curation (supporting); formal analysis (supporting). **Zhenling Shang:** Data curation (supporting); formal analysis (supporting); methodology (supporting); visualization (supporting). **Nianxue Wang:** Project administration (supporting); resources (supporting); validation (supporting). **Long Li:** Data curation (supporting); formal analysis (supporting); visualization (supporting). **Rui Zhang:** Data curation (supporting); formal analysis (supporting). **Wei Zhang:** Funding acquisition (equal); investigation (equal); methodology (equal). **Jian Zhang:** Conceptualization (equal); data curation (supporting); resources (lead); supervision (equal); writing – review and editing (equal). **Zhu Zeng:** Conceptualization (equal); data curation (equal); funding acquisition (equal); investigation (equal); methodology (equal); resources (equal); supervision (equal). **Jieheng Wu:** Conceptualization (equal); data curation (equal); funding acquisition (equal); investigation (equal); methodology (equal); resources (equal); supervision (equal).

## FUNDING INFORMATION

This work was supported by the National Natural Science Foundation of China (Grant Nos. 82160566, 12132006, 32371373), the National Natural Science Foundation of China Cultivation Program of Guizhou Medical University (21NSFCP26), the Flexible Talent Introduction Project of Guizhou Medical University (RN2021‐GK045) and Project of the Jilin Provincial Department of Education's Basic Research Program (JT53101022010).

## CONFLICT OF INTEREST STATEMENT

The authors declare that they have no conflicts of interest.

## CONSENT

All participants provided written informed consent before the research began, and the study was authorized by the ethics committee of the same institution (approval no. 2022LL‐49).

## Supporting information


Appendix S1.

## Data Availability

The datasets used for the current study are available from the corresponding author upon reasonable request.
