# Supplementary material for: CD248‐expressing cancer‐associated fibroblasts induce non‐small cell lung cancer metastasis via Hippo pathway‐mediated extracellular matrix stiffness
Source: J Cell Mol Med. 2024 Aug 20;28(16):e70025. doi: 10.1111/jcmm.70025 (PMC11335579; doi:10.1111/jcmm.70025)
Supplement: Supplementary file 1 — Appendix S1. [file JCMM-28-e70025-s001.docx]

**Supplementary Table S1**

**Primer sequence for q-PCR**

| gene name | Forward primer: | Reverse primer: |
| --- | --- | --- |
| human  CD248 | 5’-ACTACGTTGGTGGCTTCGAG-3’ | 5’-CACTGAGGAGTGGTAGGGGA-3’ |
| Human  *Anln* | 5’- ATGTCTTCGTGGCCGATTTGA -3’ | 5’- CTCTGACAGTGAGTTTCCTGTTT -3’ |
| Human  *Ankrd1* | 5’- AGCCCAGATCGAATTCCGTG -3’ | 5’- CTCCTTCTCTGTCTTTGGCGT-3’ |
| Human  *Amolt2* | 5’- GCGACTGTCAGAACAACTGC -3’ | 5’- GCACCTTTAACCTGCTTTCCA-3’ |
| Human  *Ctgf* | 5’- ACCGACTGGAAGACACGTTTG-3’ | 5’- CCAGGTCAGCTTCGCAAGG-3’ |
| Human  *Diaph1* | 5’- GTTGCAGGACCTTCGAGAGA-3’ | 5’- CCGGCACTTGAAGTCAGGAT-3’ |
| Human  *Diaph3* | 5’- GCGGTATGCATTGTAGGGGA-3’ | 5’- CAGGAGATGTAACCAGGGCA-3’ |
| Human  *Sdpr* | 5’- AAGTGCTCATCTTCCAGGAGGAAA-3’ | 5’- ATCTGAGGAGAGGTCCACGG-3’ |
| Human  *Thbs1* | 5’- AGACTCCGCATCGCAAAGG-3’ | 5’- TCACCACGTTGTTGTCAAGGG-3’ |
| Human  *GAPDH* | 5’-GGAGCGAGATCCCTCCAAAAT-3’ | 5’-GGCTGTTGTCATACTTCTCATGG-3’ |
